# Recent advances in disseminated intravascular coagulation: endothelial cells and fibrinolysis in sepsis-induced DIC

**DOI:** 10.1186/s40560-015-0075-6

**Published:** 2015-02-19

**Authors:** Seiji Madoiwa

**Affiliations:** Department of Clinical Laboratory Medicine, Tokyo Saiseikai Central Hospital, 1-14-17, Mita, Minato-ku, Tokyo 108-0073 Japan; Department of Clinical Laboratory Medicine, Jichi Medical University, 3311-1, Yakushi-ji, Shimotsuke, Tochigi 329-0498 Japan

**Keywords:** Disseminated intravascular coagulation, Endothelial cells, Fibrinolysis, Leukocyte elastase, Plasminogen activator inhibitor-1

## Abstract

Endothelial cells are highly active, sensing and responding to signals from extracellular environments. They act as gatekeepers, mediating the recruitment and extravasation of proinflammatory leukocytes to the sites of tissue damage or infection. Endothelial cells participate in fibrinolysis by secreting tissue-type plasminogen activator, which converts plasminogen to active enzyme plasmin through constitutive and regulated pathways. Fibrinolysis systems and inflammation are tightly linked, as both responses are major host defense systems against both healing processes of tissue repair as well as pathogenic microorganisms. Endothelial cell dysfunction is one of the early signs of systemic inflammation, and it is a trigger of multiple organ failure in sepsis. The marked increase in plasminogen activator inhibitor-1 level causes fibrinolytic shutdown in endotoxemia or sepsis and is one of the most important predictors of multiple organ dysfunction during sepsis-induced disseminated intravascular coagulation (DIC). Leukocytes exhibit the first-line response to microorganisms. Leukocyte-derived elastase degrades cross-linked fibrin to yield molecular species distinct from those generated by plasmin. The alternative systems for fibrinolysis that interact with the plasminogen activator-plasmin systems may play crucial roles in the lysis of fibrin clots in sepsis-induced DIC.

## Introduction

The vascular endothelium is highly dynamic and exhibits functional complexity. It performs its essential function of regulating systemic blood flow by finely tuning the diameter of blood vessels and regulates vascular tone by releasing vasodilators including nitric oxide and prostaglandin I_2_ as well as vasoconstrictors such as endothelin and platelet-activating factor. Uncontrolled and spreading systemic inflammatory responses to microbial infections play critical roles in the pathogenesis of sepsis. Under these conditions, leukocyte accumulation damages the endothelial cells and heightens permeability, leading to tissue edema.

The systemic inflammatory response occasionally causes abnormalities related to blood coagulation and fibrinolysis, ranging from subtle activation of coagulation and fibrinolysis systems to disseminated intravascular coagulation (DIC), which is characterized by simultaneous widespread microvascular thrombosis and profuse hemorrhaging [1]. However, the pathophysiology of sepsis-induced DIC is extremely complex and under extensive investigation [2]. The key event underlying life-threatening complications is the overwhelming host inflammatory response to the infectious microorganism, leading to the overexpression of inflammatory mediators [3]. This article briefly summarizes the current knowledge of the pathogenesis of DIC focusing on vascular endothelial cells and the plasminogen activator-plasmin systems in sepsis.

## Review

### Endothelial cell functions in physiological states

**Figure 1:**
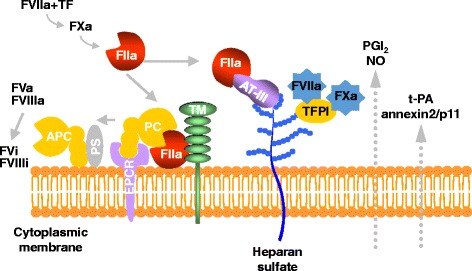
**Schematic representation of endothelial functions in physiological states.** FVIIa, activated factor VII; FVi, inactivated factor V; TF, tissue factor; AT-III, antithrombin-III; TFPI, tissue factor pathway inhibitor; PC, protein C; APC, activated protein C; PS, protein S; TM, thrombomodulin; EPCR, endothelial protein C receptor; NO, nitrate oxide; PGI_2_, prostaglandin I_2_.

Endothelial cells form the inner lining of the vascular endothelium and act as a selective barrier, controlling the trans-cellular exchange of fluids, ions, and bioactive molecules between circulating blood and perivascular tissues [4]. Under typical physiological conditions, endothelial cells actively sense and respond to signals around from their extracellular environments. Endothelial cells regulate hemostatic balance by site-specific release of procoagulants and anticoagulation factors. Endothelial cells synthesize von Willebrand factor (VWF) and protease-activated receptors (PARs) for hemostasis. They also express many molecules involved in the control of platelet function, and blood coagulation systems, including prostacyclin, nitric oxide, tissue factor pathway inhibitor, heparin sulfate, thrombomodulin, and endothelial protein C receptor (EPCR) (Figure 1) [5]. In particular, thrombomodulin-mediated binding to thrombin efficiently converts protein C to activated protein C, which results in limited inactivation of coagulation factors Va and VIIIa by proteolysis, in the presence of cofactor protein S [6]. The generation of activated protein C is accelerated by binding to EPCR and by presentation to the thrombin-thrombomodulin complex. The thrombin-thrombomodulin complex plays an indirect role in the suppression of fibrinolysis through the activation of an inhibitor, thrombin activatable fibrinolysis inhibitor (TAFI) [7]. Endothelial cells participate in fibrinolysis by secreting tissue-type plasminogen activator (tPA), which converts plasminogen to active enzyme plasmin through constitutive and regulated pathways [8,9]. This reaction is controlled by plasminogen receptors including α-enolase, histone H2B, plasminogen receptor, and annexin A2/S100A10 at the surface of endothelial cells [10,11]. The annexin A2 anchors S100A10 on the surface of endothelial cells, which recruits tPA and plasminogen to the carboxyl-terminal lysine residues of the receptor, resulting in enhanced activation of plasminogen by tPA to generate fibrinolytic activity [12]. Ectopic expression and overexpression of annexin A2 lead to uncontrolled production of plasmin, which causes fatal hemorrhagic diatheses due to hyperfibrinogenolysis in patients with malignancy [13,14].

Endothelial cells act as gatekeepers to mediate the recruitment and extravasation of proinflammatory leukocytes to the sites of tissue damage or infection. There is a multistep cascade in leukocyte trafficking, including rolling, firm adhesion, and transmigration. Each step is enhanced by the upregulation of adhesion molecules on the surface of endothelial cells and the expression of chemokines. In brief, rolling of leukocytes on the endothelial cells is promoted by binding between the endothelial E-selectin and P-selectin with specific leukocyte ligands, CD44, E-selectin ligand-1 (ESL-1), and β_2_ integrins. Firm adhesion and resistance to dislodgement by fluid shear stress is promoted by the bindings of the endothelial cell protein vascular cell adhesion molecule (VCAM)-1 and intercellular adhesion molecule (ICAM)-1 with leukocyte integrins [15]. The leukocytes then transmigrate between endothelial cells into extravascular tissues, which requires the coordinated redistribution of lymphocyte function-associated antigen-1 (LFA-1) and endothelial cell ICAM-1.

### Fibrinolysis under normal physiological conditions

Following the activation of blood coagulation systems, fibrin generation and tissue hypoxia stimulate an increase in tPA secretion from endothelial cells, which initiates and accelerates the conversion of plasminogen to active enzyme plasmin [16]. Plasmin is a serine protease that hydrolyzes cross-linked fibrin, resulting in the release of a variety of complexes made up degradation products. The DD/E fragment is the smallest complex, consisting of two fragment D moieties and a non-covalently bound fragment E. DD/E is held together by uncleaved coiled coils, with larger forms representing longitudinally associated strings of DD/E.

Several types of cells, including vascular endothelial cells, produce plasminogen activator inhibitor-1 (PAI-1), which specifically regulates the initial phase of fibrinolysis by inhibiting plasminogen activators. PAI-1 efficiently inhibits tPA by rapid binding, forming a stable 1:1 complex. PAI-1 acts as an acute phase reactant, as its levels drastically increase during various inflammatory responses [17]. α_2_-Plasmin inhibitor (α_2_-PI) is a physiological inhibitor of plasmin that regulates the late phase of fibrinolysis. α_2_-PI circulates in plasma at half the molar concentration of plasminogen. Thus, the α_2_-PI concentration is sufficient to neutralize plasmin under normal physiological conditions, where only small amounts of plasminogen are activated. α_2_-PI rapidly inhibits plasmin by irreversibly binding, forming an inactive complex with plasmin (plasmin-inhibitor complex; PIC). In addition, α_2_-PI inhibits the binding of plasminogen to fibrin molecules through competition for lysine binding sites, which is a unique regulatory phenomenon of fibrinolysis. During stable clot formation, α_2_-PI is cross-linked to fibrin by factor XIIIa, and α_2_-PI-bound fibrin is resistant to degradation by plasmin. TAFI is a carboxypeptidase B-like proenzyme that is converted to active enzyme TAFIa by thrombin. The endothelial cell receptor thrombomodulin stimulates the thrombin-mediated TAFI activation greater than 1,000-fold [7]. TAFIa slowly inhibits the fibrinolytic processes by removing carboxyl-terminal lysine residues from fibrin molecules. This action is inhibitory because carboxyl-terminal lysine residues are important stimulators, serving as binding sites for tPA and plasminogen, as well as plasmin.

### Inflammation and endothelial cells

Blood coagulation and inflammation are tightly linked, as both responses are major host defense systems of the healing process of damaged tissue repair and against the presence of pathogenic microorganisms [18]. Tissue damage and exposure to proinflammatory stimuli shift the balance of the endothelial function toward a procoagulant phenotype [19]. Inflammation is characterized by increased permeability of the vascular endothelium, causing leakage of blood components and extravasation of immune cells, including macrophages and dendritic cells [20]. The vascular endothelial cells contribute to innate immunity as nonprofessional effectors. These cells recognize the presence of microorganisms through their toll-like receptors (TLRs), which are responsible for recognizing structures conserved among microbial species, pathogen-associated molecular patterns (PAMPs). Sepsis is associated with a dysfunction of the host immune system response to invading pathogens [21]. An excessive response to TLR ligands is known to induce lethal septic shock. TLRs are also capable of detecting endogenous molecules released from damaged cells, termed as damage-associated molecular patterns (DAMPs). Thus, stimulations of PAMPs or DAMPs through TLRs activate signaling pathways that depend mainly on MyD88 and TRIF, leading to the translocation of NF-κB and IRFs into the nucleus [20]. The activation of these transcription factors induces the expression of type I interferons (IFNs) and proinflammatory cytokine genes such as tumor necrosis factor (TNF) and IL-6.

The coagulation serine proteases initiate proinflammatory or anti-inflammatory signaling through PARs, which are widely expressed on platelets, endothelial cells, and immune cells [22]. In endothelial cells, responses to thrombin are mediated by PAR1, and responses to tissue factor/factor VIIa and factor Xa are mainly facilitated by PAR2 [23]. PAR1 activation by thrombin stimulates Rho-dependent cytoskeletal responses involved in the permeability and migration of endothelial cells [24]. Activation of endothelial surfaces by PAR1 promotes adhesion and rolling of leukocytes. The sphigosine 1-phosphate (S1P) is a downstream component of PAR1 signaling that plays a critical role in the amplification of the inflammation response, through the regulation of motility of dendritic cells onto lymph nodes during severe sepsis [25]. Interestingly, activated protein C on EPCR elicits cytoprotective signals through the activation of PAR1, which is fundamentally different from activation by thrombin. The localized activation of PAR1 in caveolae by activated protein C on EPCR and transactivation of the S1P receptor are thought to be responsible for the anti-inflammatory and anti-apoptotic effects [26]. Moreover, TAFIa promotes anti-inflammatory effects by inactivating the complement factors C5a and C3a on endothelial cells. *In vivo* studies demonstrated that TAFI deficiency exacerbated allergic inflammation and acute lung injury [27,28].

### Dysfunction of endothelial cells in sepsis

Sepsis is a systemic inflammatory response syndrome (SIRS), occurring in patients with infection or injury. The dysfunction of vascular endothelial cells is one of the early signs of a systemic inflammation and is a trigger for multiple organ failure in sepsis [29]. The clinical features of sepsis-induced DIC include widespread thrombosis in the microcirculation of different tissues, resulting in severe disruption of organ homeostasis. The development of DIC in septic patients has been found to be an independent predictor of mortality [30].

### Endothelial cell dysfunction and regulation of blood coagulation

The vascular response against to inflammation is characterized by smooth muscle changes, inducing vascular dilation and endothelial cell contraction. These result in vascular leakage of proteins into extravascular spaces and tissues. The inflammatory provocations also promote the migration of leukocytes from the microvascular circulation into inflammatory sites by stimulating increased expression of adhesion molecules on the surface of endothelial cells, including selectins.

**Figure 2:**
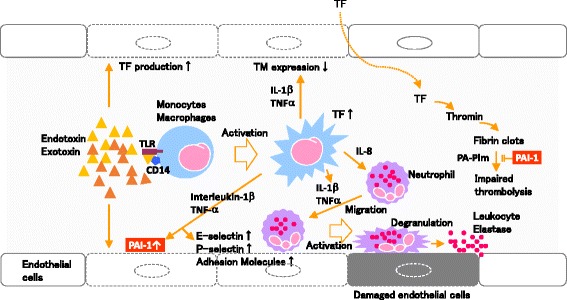
**Schematic representation of endothelial dysfunction in sepsis.** TF, tissue factor; IL-1β, interleukin-1β; TNFα, tumor necrosis factor α; TLR, toll-like receptor; PA-Plm, plasminogen activators-plasmin system; PAI-1, plasminogen activator inhibitor-1.

The blood coagulation system is drastically altered in sepsis, as the physiological hemostatic balance is shifted toward a procoagulation state. Tissue factor expression is induced in CD14^+^ monocytes and endothelial cells responding to acute inflammatory mediators (Figure 2). Neutralizing TNF activity by introducing a TNF receptor-IgG fusion protein, or an anti-TNF antibody, did not improve endotoxin-induced coagulopathy [31,32]. By contrast, inhibition of IL-6 completely abrogated tissue factor-dependent thrombin generation, suggesting a major role for endogenous IL-6, and to a lesser extent, IL-1 [33]. Tissue factor from endothelial cells can be shuttled between cells through microparticles derived from activated mononuclear cells [34]. However, the contribution of tissue factor-positive microparticles to the development of DIC has not been determined [35].

Antithrombin is the primary inhibitor of both thrombin and factor Xa and is markedly decreased in sepsis. This decrease is caused by the reduction in hepatic synthesis of the negative acute phase protein, consumption by formation of thrombin-antithrombin complexes, and degradation by proteases released from activated neutrophils [36]. In addition, glycosaminoglycans including heparin sulfate, hyaluronic acid, and chondroitin sulfate form an endothelial surface layer, which contributes to the regulation of thrombin and factor Xa by inducing the allosteric activation of antithrombin III. Degradation of the endothelial surface layer has been implicated in the pathogenesis of systemic inflammation, and protection of glycosaminoglycans is a goal of resuscitation strategies for intensive care unit (ICU) patients [37]. Much attention has been devoted to the role of thrombomodulin as a pivotal determinant of endothelial thrombo-resistance as well as a target for the resolution of vascular disorders in DIC. Suppression of endothelial thrombomodulin expression by TNF-α has been documented [38]. Several studies have suggested that neutrophil-derived elastase and cathepsin G proteolytically cleave endothelial cell surface thrombomodulin. This increased thrombomodulin shedding is a potential causative element of vascular injury [39]. An elevated thrombomodulin serum level is widely regarded as an important biomarker for endothelial dysfunction [40] and has been found to correlate with the severity of sepsis-induced DIC [41]. In addition, the amino-terminal lectin-like domain of thrombomodulin plays a critical role in regulating inflammatory responses. This domain functions by inhibiting leukocyte adhesion onto endothelial cells and complement pathways and degrading proinflammatory high-mobility group box 1 protein [42]. The therapeutic benefits of administering recombinant soluble thrombomodulin as a treatment for severe inflammatory disorders such as sepsis-induced DIC have been demonstrated in studies conducted in Japan [43].

### Endothelial cell dysfunction and the fibrinolysis system

**Figure 3:**
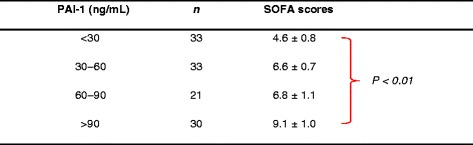
**Plasma plasminogen activator inhibitor-1 (PAI-1) levels correlate with multiple organ dysfunction scores using the sequential organ failure assessment (SOFA) score in sepsis-induced DIC patients.** DIC patients were classified into four groups according to PAI-1 levels at the time of DIC diagnosis (<30 ng/mL, 30–60 ng/mL, 60–90 ng/mL, and >90 ng/mL), and the groups were compared with respect to SOFA scores. Data are presented as the mean ± SEM. Reprinted with modifications from International Journal of Hematology [46].

The fibrinolysis system is mainly mediated by plasminogen activators-plasmin system and regulated by a principal inhibitor PAI-1. Numerous studies have consistently found that a marked increase in PAI-1 results in a fibrinolytic shutdown in endotoxemia or sepsis, although a simultaneous increase in tPA often occurs [44]. Indeed, fibrinolysis is suppressed by increased PAI-1 levels in the plasma of DIC patients exhibiting SIRS with sepsis or trauma. Although there may be some fibrinolytic activity in response to the extensive formation of fibrin, the levels of this activity are too low to counteract the systemic deposition of fibrin clots in SIRS. PAI-1 expression was localized primarily to endothelial cells at all levels of the vasculature in endotoxemic animal models, suggesting that plasma PAI-1 originates from endothelial cells [45]. High levels of PAI-1 have been implicated as predictive for an adverse outcome in severe sepsis, and suppressed fibrinolysis is one of the most important predictors of multiple organ dysfunction during sepsis-induced DIC (Figure 3) [46]. Additional measurements were made at the time of ICU admission independently discriminated between patients who developed DIC from those who did not, including increased levels of PAI-1, as well as thrombin-antithrombin complex, a biomarker for activation of coagulation, and decreased protein C as an indicator for consumption of coagulation factor [47].

Transient activation of plasminogen activators-plasmin systems has been observed during the immediate posttraumatic period, which results in a state of shock with serious hemorrhaging [48]. Less than 1 h after severe injury, catecholamine-induced endothelial damage, hypoxia, and ischemia may invoke a marked release of tPA from the endothelium into circulation resulting in the conversion of large amounts of plasminogen to plasmin [49]. However, there is some controversy and conflicting data still surround the exact underlying pathophysiology. Following the 24–48 h after trauma, the inflammatory influences on coagulation favor a more prothrombotic tendency, where proinflammatory cytokines downregulate protein S, endothelial-based heparin sulfate proteoglycans, thrombomodulin, and endothelial protein C receptor.

### Endothelial cell dysfunction and leukocyte elastase

The release of PAMPs during a microbial infection induces tissue macrophages to generate inflammatory cytokines, including TNF-α, IL-1, and IL-8. The localized activation of these cytokines contributes to host defenses by attracting activated leukocytes to the site of infection. However, the entry of inflammatory cytokines and PAMPs into blood circulation may lead to microvascular damage [50]. Leukocytes are known to release enzymes with intrinsic proteolytic activity, including leukocyte elastase and cathepsin G, in a variety of clinical conditions. The subsequent scope of leukocyte elastase activity is mediated by endogenous inhibitors, predominantly α_1_-protease inhibitor [51,52]. Leukocyte elastase is a potent protease, as it cleaves nearly all connective tissue components, including elastin and a variety of proteoglycans. Interestingly, leukocyte elastase has two opposing roles in fibrinolysis [53]. It promotes fibrinolytic functions by degrading fibrinogen and fibrin and inactivating PAI-1 [54]. By contrast, leukocyte elastase has antifibrinolytic activities cleaving plasminogen activators and plasmin [55].

Microthrombi caused by endothelial injuries induce ischemia and cause damage to a variety of organs in sepsis-induced DIC. Multiple studies have implicated the proteases released from leukocytes to be involved in the progression of multiple organ injuries. A deficiency of ADAMTS13, also known as VWF-cleaving protease, leads to the presence of unusually large multimers. These are responsible for the aggregation of platelet formation of microthrombi in the circulatory system, which leads to typical thrombotic microangiopathies [56]. Cleavage of ADAMTS13 by leukocyte elastase together with thrombin and plasmin cause a severe secondary ADAMTS13 deficiency, and there may be a clinical correlation with renal endothelial cells dysfunction in patients with sepsis-induced DIC [57].

Leukocytes exhibit the first line of response to microorganisms. Activated leukocytes and dying cells release histones (particularly histones H3 and H4) and may promote thrombus formation by inducing endothelial injury during sepsis [58]. In addition, neutrophils stimulated by activated platelets release the neutrophil extracellular traps (NETs) containing histones, DNA, and leukocyte-derived proteases [59]. Although NETs have a pivotal role in the suppression of pathogen dissemination and killing of microorganisms, they also induce tissue damage and thrombus formation [60,61].

**Figure 4:**
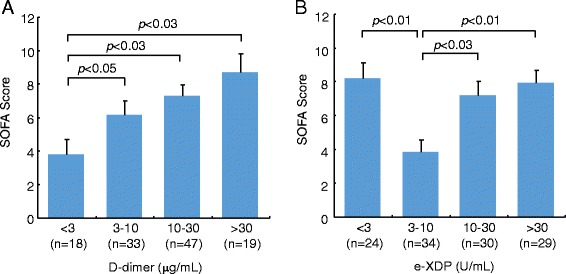
**Relationships between the SOFA score and cross-linked fibrin degradation product by leukocyte elastase (e-XDPs) levels in sepsis-induced DIC patients.** Patients with sepsis-induced DIC were classified into four groups with e-XDPs levels (<3 U/mL, 3–10 U/mL, 10–30 U/mL, >30 U/mL) at the time of DIC diagnosed, and the groups (**(A)** D-dimer and **(B)** e-XDP) were compared with respect to SOFA scores. Data are presented as means ± SEM. Reprinted with modifications from Thrombosis Research [66].

Exposure to inflammatory mediators and interaction with leukocytes causes endothelial activation and dysfunction, directly or indirectly [62]. However, leukocyte elastase has been found to degrade cross-linked fibrin and to yield molecular species distinct from those generated by plasmin [63]. Thus, leukocyte elastase-mediated fibrinolysis is activated to varying degrees, depending on the extent of systemic inflammation (Figure 4) [64]. These alternative systems for fibrinolysis, comprised of proteases other than plasmin and their interactions with the plasminogen activators-plasmin systems, have been thought to play crucial roles in the fibrin lysis of clots in sepsis-induced DIC [65,66]. The evaluation of leukocyte elastase-mediated fibrinolysis and regulation of its activity by specific inhibitors could improve the poor outcomes common to patients with sepsis-induced DIC [67].

## Conclusions

It is clear that endothelial cell function, inflammatory mediators, and the associated crosstalk with blood coagulation and fibrinolysis systems are altered in sepsis-induced DIC and that significant heterogeneity exists in the host response against a microbial infection [68]. Further research elucidating basal mechanisms at the cellular and molecular levels may bring clinicians closer to improving morbidity and mortality of patients with sepsis-induced DIC.
